# Inhibition of Mitochondrial Complex I Impairs Release of α-Galactosidase by Jurkat Cells

**DOI:** 10.3390/ijms20184349

**Published:** 2019-09-05

**Authors:** Jonathan R. A. Lambert, Steven J. Howe, Ahad A. Rahim, Derek G. Burke, Simon J. R. Heales

**Affiliations:** 1 Enzyme Unit Great Ormond Street Hospital, London WC1N 3JH, UK; 2 University College London Great Ormond Street Institute of Child Health London, London WC1N 1EH, UK; 3 University College London School of Pharmacy, University College London, London WC1N 1AX, UK; 4 Neurometabolic Unit, National Hospital, London WC1N 3BG, UK

**Keywords:** mitochondria, Fabry, cross correction

## Abstract

Fabry disease (FD) is caused by mutations in the *GLA* gene that encodes lysosomal α-galactosidase-A (α-gal-A). A number of pathogenic mechanisms have been proposed and these include loss of mitochondrial respiratory chain activity. For FD, gene therapy is beginning to be applied as a treatment. In view of the loss of mitochondrial function reported in FD, we have considered here the impact of loss of mitochondrial respiratory chain activity on the ability of a *GLA* lentiviral vector to increase cellular α-gal-A activity and participate in cross correction. Jurkat cells were used in this study and were exposed to increasing viral copies. Intracellular and extracellular enzyme activities were then determined; this in the presence or absence of the mitochondrial complex I inhibitor, rotenone. The ability of cells to take up released enzyme was also evaluated. Increasing transgene copies was associated with increasing intracellular α-gal-A activity but this was associated with an increase in Km. Release of enzyme and cellular uptake was also demonstrated. However, in the presence of rotenone, enzyme release was inhibited by 37%. Excessive enzyme generation may result in a protein with inferior kinetic properties and a background of compromised mitochondrial function may impair the cross correction process.

## 1. Introduction

Fabry disease (FD) is a sphingolipidosis associated with cellular accumulation of globotriaoslyceramide (Gb3) [1] caused by deficiency in α-galactosidase A (α-gal-A) (EC 3.2.1.22) [2]. The enzyme hydrolyses terminal galactose from Gb3 to produce lactosylceramide [3]. This is an essential step in the sequential catalysis of globoside to ceramide within lysosomes [4]. The gene encoding α-gal-A, identified as *GLA* (NCBI: NM_000169.2), was mapped to the long arm of the X chromosome (Xq21-22) [5], and the coding regions of the gene were sequenced for the mature lysosomal form of the human enzyme [6]. 

Though a single gene disorder, the phenotypic consequences of pathogenic mutations in *GLA* are heterogeneous, multi-systemic and generally progress to affect cerebrovascular, cardiovascular and/or renal systems. There are currently two forms of treatment available to patients: enzyme replacement therapy (ERT) [7,8] and small molecule chaperone therapy [9]. Whilst ERT has been shown to benefit some patients, there is considerable variation in levels of Gb3 clearance from tissue-types and clinical response across age and disease severity [10,11,12]. Chaperone therapy is applicable for amenable mutations which account for 35–50% of pathogenic mutations in *GLA*, so at least half of all FD patients will not benefit from this mode of treatment [9,13]. 

Alternative therapies should be considered. Substrate reduction therapy, an orally administered glucosylceramide synthase inhibitor, has recently completed its first early-stage clinical trial in patients receiving ERT [14]. Another option is to address the root cause of the disorder by inserting a copy of the cDNA for α-gal-A into patient cells to enable synthesis of functional α-gal-A for long-term therapeutic benefit. This is the rationale behind gene therapy [15]. 

With regards to FD, a recombinant lentivirus approach targeted to ex-vivo haematopoietic stem cells (HSC) is currently being evaluated in a human trial [16]. Data supporting the trial, utilizing an animal model, revealed enzyme correction in a number of tissues including leukocytes and plasma [16,17]. Whilst such results are clearly encouraging, it is possible that other factors might need to be considered. When considering treatment options for FD, and other lysosomal disorders, an optimal therapeutic outcome will be dependent on cross correction, i.e., transfer of enzyme from replete (corrected) to deficient cells [18,19,20,21]. In FD, disease loss of metabolic homoeostasis, due to substrate accumulation, may negatively impact on process such as energy metabolism [22,23,24]. This may arise as a consequence of disruption of mitochondrial function due to a failure of mitophagy [25,26], oxidative stress [27,28,29], and membrane lipid imbalance [30]. Consistent with this, reduction in the activity of respiratory chain complexes has been reported in FD fibroblasts [31]. This may in turn lead to low ATP availability and/or increased oxidative stress [32]. Concerning ATP, this has been reported to be required to facilitate lysosomal enzyme release from polymophonuclear leukocytes [33]. Furthermore, increased oxidative stress may also impair enzyme release [34,35,36]. Together, this raises the research question: does a background of loss of mitochondrial respiratory chain activity affect the ability of cells to cross-correct?

In this study, we have used Jurkat cells, a T-lymphoblastic leukaemia cell-line. These cells exhibit low α-gal-A activity relative to other cells [37] and are therefore useful as a proof of principle cell line to evaluate α-gal-A overexpression and cross correction in the presence and absence of a compromised mitochondrial respiratory chain.

## 2. Results

### 2.1. Dose-Dependent Increase in Intracellular α-galA Activity with Transgene Overexpression 

**Figure 1:**
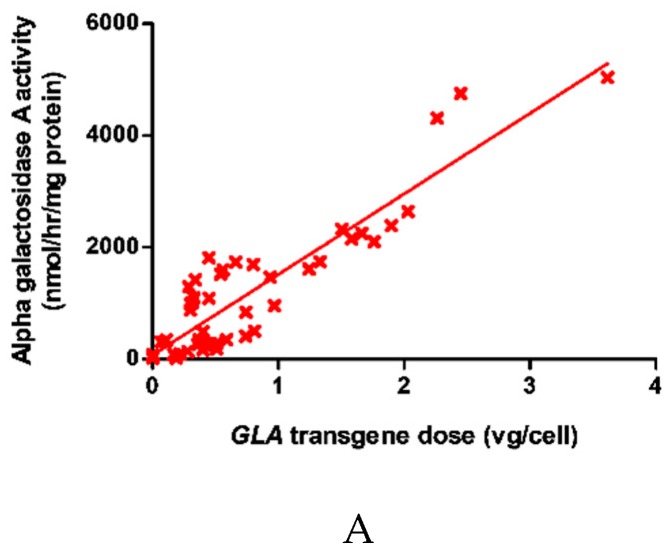

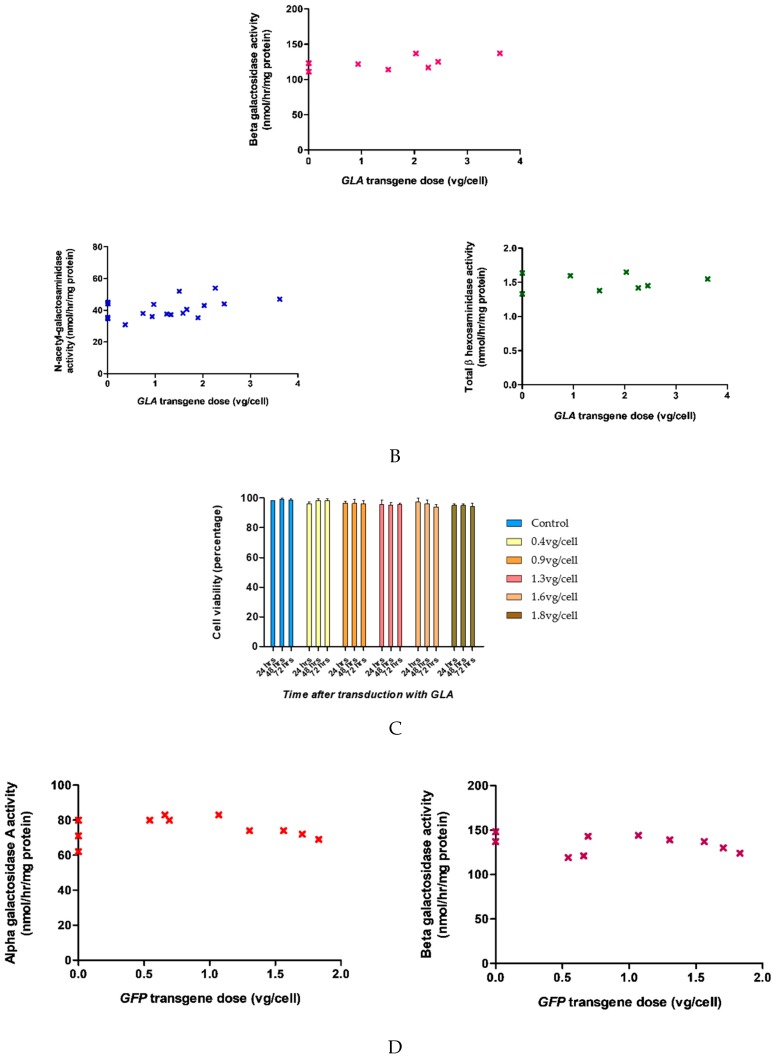
Increase in intracellular α-gal-A activity following transfection and with no effect on other lysosomal hydrolases or cell viability. Intracellular α-gal-A activity increased with GLA transgene dose. Linear regression R^2^ = 0.835, *p* < 0.0001 (**A**). The activity of three other lysosomal enzymes was measured and shows no correlation with virus dose (**B**). Cell viability showed no evidence of cell toxicity following transduction. (**C**). Activity of α-gal-A and β-galactosidase in Jurkats transduced with lentivirus containing *GFP* did not correlate with virus dose (**D**).

α-gal-A activity was found to increase linearly with *GLA* copy number within the cells as shown in Figure 1A. In order to ascertain specificity for *α-galA,* the activity of three other lysosomal enzymes was determined. There was no relationship between *GLA* transgene dose and the activity of β-galactosidase, total β-hexosaminidase or N-acetyl galactosaminidase (Figure 1B). Concerning cell viability, there was no evidence of cell toxicity (trypan blue exclusion) up to 1.8 vg/cell (Figure 1C). Finally, neither α-gal-A nor β-galactosidase activity correlated with virus dose in Jurkats transduced with the *GFP* transgene within the same lentivirus backbone (Figure 1D).

### 2.2. α-Gal-A Activity and Michaelis Constant Increased in Cells Following Transduction with The GLA Transgene at between 0.4 vg/cell and 1.5 vg/cell

Increased viral dose was found to result in an increase in maximum velocity (Vmax) and K_m_ (decreased affinity) as shown in Table 1.

At 0.4 vg/cell, Vmax increased and the Km was comparable to the wild-type cells. At 1.5 vg/cell, Km significantly (*p* < 0.01) increased as did Vmax. At 2.0 vg/cell, Vmax again increased whilst Km was comparable to that reported at 1.5 vg/cell. To evaluate further the effect upon Km, homogenates prepared from cells exposed to 1.8 vg/cell were diluted down to provide a Vmax comparable to that reported in wild-type cells. Under these conditions, Km was still found to be significantly (*p* < 0.01) elevated when compared to the wild-type cells. 

### 2.3. Release of α-Gal-A into Surrounding Growth Media by GLA-Jurkats Was Both Dose- and Time-Dependent, without Evidence of Cellular Damage 

**Figure 2:**
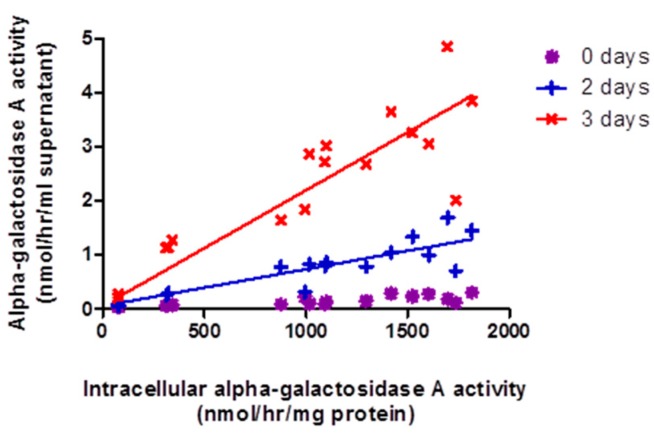
Release of α-gal-A by *GLA* Jurkats. A-gal-A activity was measured in the culture media supernatant collected after zero, two and three days incubation at 37 °C with *GLA*-Jurkats containing between zero and 0.5 vg/cells. The extracellular activity in supernatant was plotted against intracellular activity within the *GLA*-Jurkats. Secretion of α-gal-A activity into the surrounding media by *GLA*-Jurkats increased with both intracellular activity and duration of incubation. Linear regression found that at day 0, R^2^ = 0.607 (*p* < 0.001); day 2, R^2^ = 0.786 (*p* < 0.0001); and day 3, R^2^ = 0.796 (*p* < 0.0001).

α-gal-A activity in the bathing cell culture media was found to be dependent upon the intracellular enzyme activity of the transduced cells and the number of days of incubation (Figure 2). No secretion of total β-hexosaminidase was observed up to 2 days, suggesting that release was specific to α-gal-A and not due to cell damage.

### 2.4. Wild-Type Jurkats Grown in Media Overexpressing α-Gal-A Showed Increased Intracellular α-galA Activity, Consistent with Enzyme Uptake from the Media 

**Figure 3:**
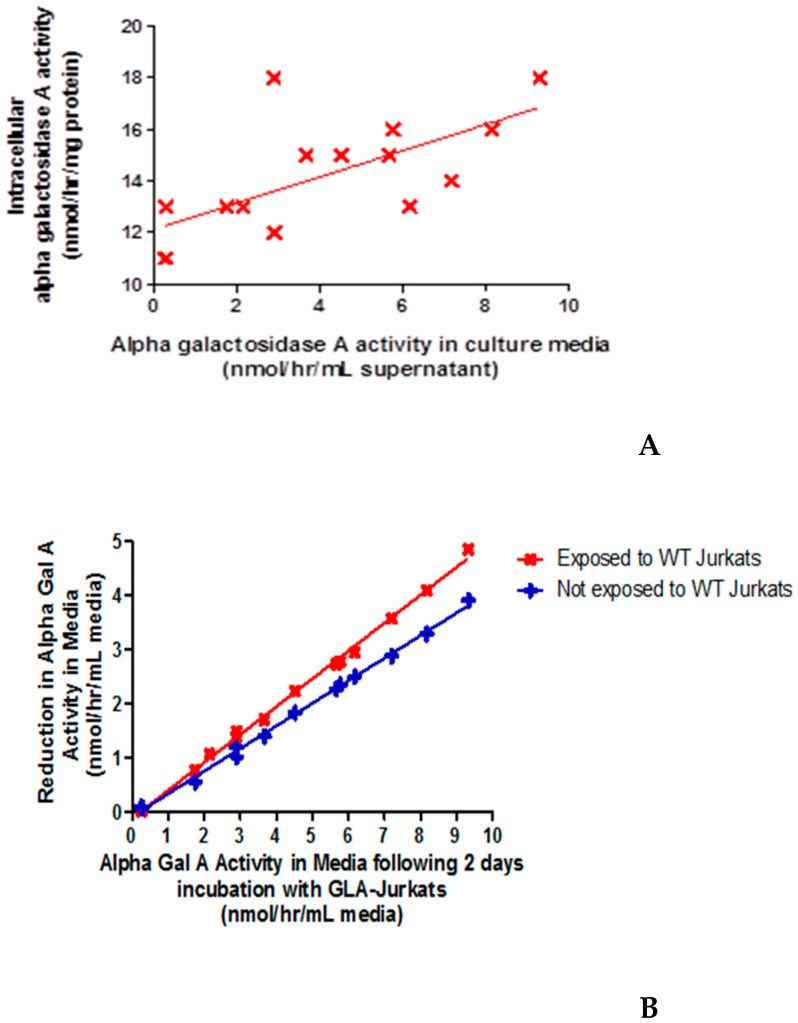
Uptake of alpha-galactosidase A activity by wild-type naive Jurkats. (**A**). Bathing (donor) media, from over expressing α-gal-A Jurkats, were taken after two days in culture. Recipient cells (wild-type Jurkats) were incubated for two days in the donor media. Intracellular α-galA was plotted against the initial α-gal-A activity in the donor media. There was significant increase in α-gal-A activity in the recipient cells with increasing donor media activity. Linear regression R^2^ = 0.450 (*p* < 0.01) (**B**). Comparison of the reduction in α-galA activity in donor media with and without cells present. There was a significant difference between the slopes of the lines (*p* < 0.0001), consistent with take-up of enzyme from donor media to recipient cells.

Figure 3A shows that intracellular α-gal-A activity within the recipient cells increased with initial activity within the donor media. This was not accompanied by any change in intracellular total β-hexosaminidase activity. In addition, qPCR confirmed no contamination of the wild-type Jurkats with lentivirus (data not shown). These results suggest uptake of functional enzyme by the wild-type recipient cells from the donor media. Consistent with this, extracellular α-gal-A activity decreased within donor media following incubation with recipient cells for two days. Degradation in enzyme activity within the culture media was observed, but the rate of decline in activity in culture media was significantly greater (*p* < 0.0001) when incubated with wild-type Jurkats, i.e., further suggesting enzyme uptake by the cells (Figure 3B).

### 2.5. Rotenone Treatment Resulted in Marked Loss of Complex I Activity in Jurkat Treated Cells

**Figure 4:**
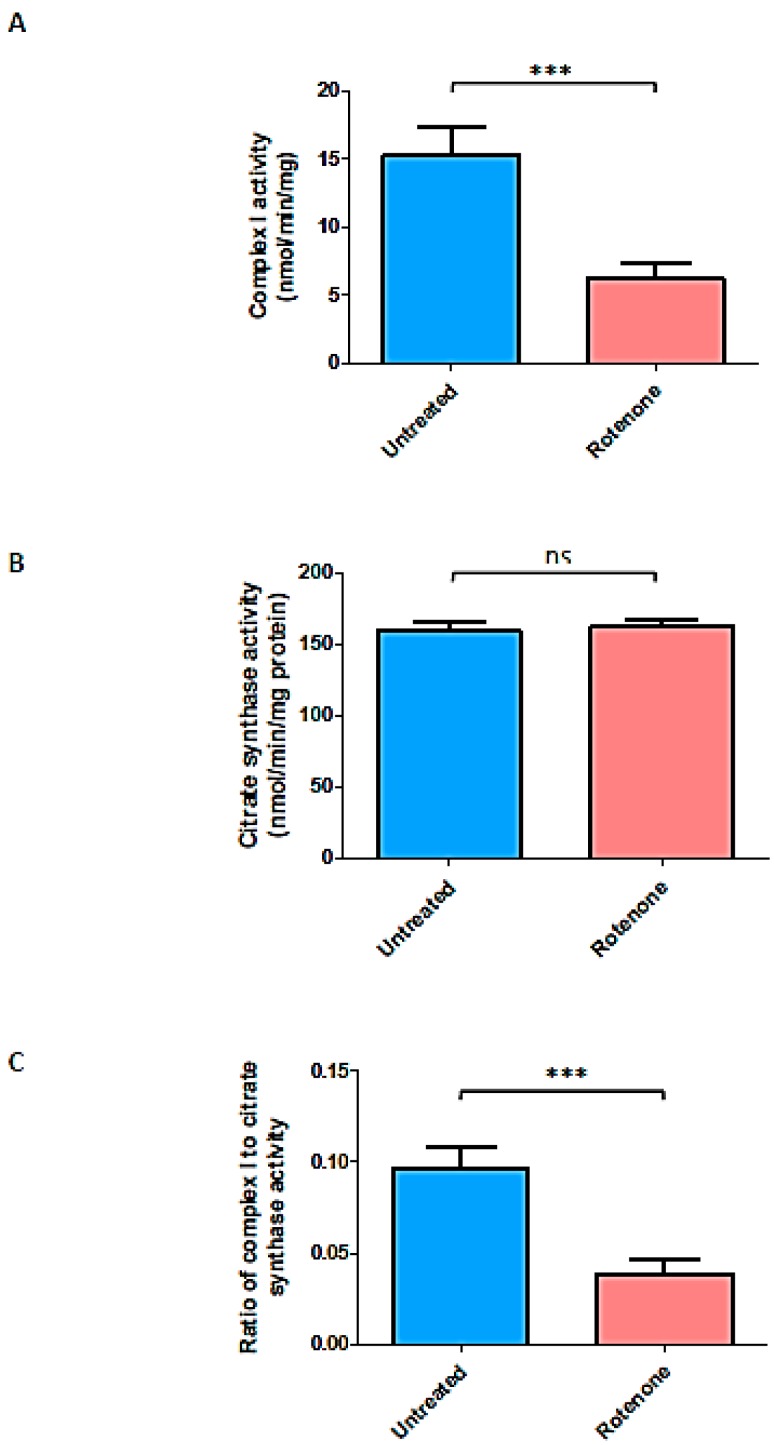
The effect of rotenone on mitochondrial complex I activity in wild-type Jurkats. An amount of 100 nM rotenone significantly (*** *p* < 0.0001) decreased complex I activity (protein baseline) when compared with untreated controls (**A**). There was no significant (ns) effect on citrate synthase activity (**B**). The ratio of complex I activity to citrate synthase also showed a significant (*** *p* < 0.0001) reduction in the presence of rotenone (**C**).

Treatment of Jurkat cells with rotenone was found to inhibit complex I by 60% without affecting mitochondrial content, as judged by citrate synthase activity (Figure 4). 

### 2.6. Rotenone Treatment Did Not Significantly Affect Transduction Efficiency of The Lentivirus Containing GLA Transgene

**Figure 5:**
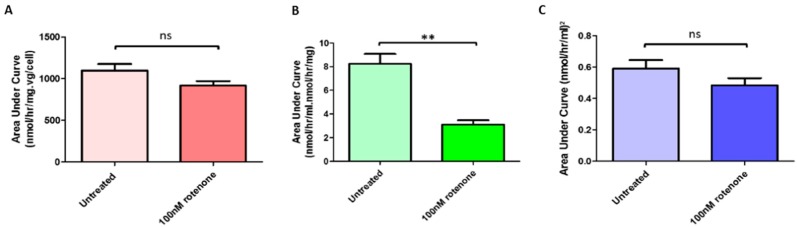
Effect of mitochondrial complex I inhibition on (**A**) lentivirus-mediated transduction efficiency, (**B**) enzyme release (** *p* < 0.001) and (**C**) enzyme uptake.

In the presence of the complex I inhibitor, intracellular α-gal-A activity was not significantly affected (Figure 5A) when incubated with the lentivirus containing the GLA transgene. Furthermore, since, under the conditions employed, enzyme activity was unaffected this implies that rotenone had no direct effect upon the α-gal-A assay. 

### 2.7. Rotenone Treatment Significantly Reduced Release by GLA-Jurkats into the Surrounding Media

α-gal-A activity was determined in the extracellular media derived from the above experiments. Figure 5B shows that rotenone treatment significantly reduced the amount of enzyme activity released into the media from the cells. There was no effect of virus dose or rotenone treatment on the activity of total β-hexosaminidase in the culture media.

### 2.8. Rotenone Treatment Did Not Significantly Alter Uptake of α-Gal-A by Wild-Type Recipient Jurkats from Donor Media Taken from Transduced Jurkats

Disappearance of α-gal-A activity from the media derived from above was determined in the presence of naive wild-type *Jurkat* cells. Figure 5C shows that there was no statistically significant difference in uptake from the media by the cells treated with and without 100 nM rotenone for 24 h.

**A**: Jurkats were transduced with the lentiviral vector at doses from zero to 0.4 vg/cell; this in the presence or absence of rotenone. After 2 days, intracellular enzyme activity was plotted against viral dose and the area under the curve calculated, i.e., to record the total enzyme activity for the performed incubations. There was no significant (ns) difference in total α-gal-A activity in the presence and absence of rotenone. 

**B**: Extracellular α-gal-A activity in bathing media from the above experiment was determined. Activity was plotted against original viral dose and area under the curve determined. A significant (** *p* < 0.001) reduction in total extracellular enzyme activity was recorded for the cells treated with rotenone. 

**C**: Uptake of enzyme from conditioned cell culture media (from experiment A) by naive wild-type (recipient) Jurkats was determined by assessing the disappearance of α-gal-A activity from the cell culture medium. Activity was plotted against original viral dose and area under the curve calculated. There was no significant difference (ns) in uptake by recipient cells with or without complex I inhibition.

## 3. Discussion

Current treatments for FD are limited. As a monogenic disorder, it is a potential candidate for gene therapy. Lentiviral vectors represent a highly efficient method of gene delivery to patient cells for long-term and stable transgene expression. 

An ex-vivo approach to FD, utilising lentivirus, is now under trail, i.e., based in part on animal studies that demonstrated increased α-gal-A activity in a number of tissues including plasma and peripheral leukocytes [16]. In this study, we have utilised Jurkat cells (T-lymphocyte cell line) as a proof of principle model to ascertain whether factors such as viral load and a background of impaired mitochondrial respiratory chain activity need to be considered. 

Transduction of Jurkats with the lentiviral vector resulted in a dose-dependent increase in intracellular α-gal-A activity, with no evidence of cytotoxicity up to 1.8 vg/cell. This finding suggests that overexpressed α-gal-A is undergoing post-translational modifications and transportation to the lysosome for maturation and activation to occur and that transduction of cells with these amounts of vector is not detrimental to cell health. This is consistent with many previous studies that showed engineered vectors containing cDNA encoding human α-gal-A can generate therapeutic *GLA* transgene expression in transduced target cells [16,17,18]. 

Our kinetic studies found that K_m_ of α-gal-A in wild-type Jurkats measured by hydrolysis of synthetic fluorogenic substrate 4MUG (K_m_ = 1.57 mM) was comparable with K_m_ seen in human plasma (K_m_ = 1.9 mM, [38]), suggesting that endogenous α-gal-A from Jurkats has similar kinetic properties from plasma. Calculation of K_m_ with increasing viral dose revealed a significant loss of affinity for the substrate (increased K_m_) at a dose of above 1.5 vg/cell. Whilst the mechanism for this apparent increase in K_m_ is not yet known, our findings suggest a potentially important need to optimize virus dose in order to deliver maximum therapeutic benefit to Fabry disease patients. This appears to be the first study exploring the kinetic properties of α-gal-A produced by transduced cells. 

Next, cross correction between transduced and treatment-naive cells was explored. *GLA*-Jurkats showed virus dose- and time-dependent release of α-gal-A activity into culture media. Media collected after two days incubation were collected and used to incubate treatment-naive Jurkats for a further two days, during which time the cells specifically took up α-galA from the media. Together, this demonstrated cross correction between Jurkats as observed previously in fibroblasts [21] and immortalized lymphoblasts derived from Fabry disease patients [18]. 

FD is known to interrupt energy metabolism in cardiomyocytes [23,24,39] and brain tissue [40]. Furthermore, the activity of mitochondrial respiratory chain complexes is reduced in skin fibroblasts from patients [31]. In view of these affects, rotenone was used to inhibit mitochondrial complex I in Jurkats to study the effect upon cross correction. 

Using this experimental approach, we found that complex I inhibition had no effect upon the transduction efficiency of the lentivirus on Jurkats. This suggests that loss of mitochondrial function in Fabry disease may not prevent cells treated with gene therapy from manufacturing therapeutic enzyme and is an encouraging finding for development of future gene delivery for patients. However, the ability of transduced Jurkats to secrete overexpressed α-galA out into their surrounding media over 24 h was reduced by 37%. Loss of complex I may impact the cell in a number of ways including decline in ATP bioavailability and increase in oxidative stress. Under the conditions employed, rotenone treatment is reported to decrease ATP levels by approximately 20% with a comparable increase in generation of reactive oxygen species [41,42]. Since there is evidence that ATP is required in the release process [43], our finding may therefore be related to compromised cellular energy metabolism. Oxidative stress and production of reactive oxygen species could further impact mitochondrial function and hence could contribute to the mechanism(s) that compromise enzyme release. [35]. Our work may also provide a potential mechanism by which cross correction is impaired in female FD patients [44]. Finally, there was no effect on uptake. 

Taken together, these findings may point to a reliance on mitochondrial function to ensure cross correction occurs at the level of release. If oxidative stress is subsequently demonstrated to be a consequence of loss of respiratory chain complex activity in FD, then antioxidant therapies could be considered as an adjunct to gene therapy, e.g., precursors of reduced glutathione and/or lipophilic antioxidants such as vitamin E (and analogues). In addition, in view of the loss of mitochondrial function associated with FD, agents that may stimulate mitochondrial function/biogenesis may also be considered in further studies, i.e., with a view of correcting an energy deficient state. However, if enzyme replacement therapy or restoration of enzyme activity by gene transfer restores mitochondrial function then cross creation could proceed. Further work is now required to test these hypotheses.

## 4. Materials and Methods

### 4.1. Jurkat Cells 

Jurkat cells (TIB-152) were obtained from the American Type culture collection (ATCC, Teddington, UK).

### 4.2. Lentiviral Vector Production

HIV-1-based lentivirus vectors were generated by transfection of 293T cells (ATCC, Teddington, UK) with vector plasmid *pRRLSIN.cPPT.PGK-GLA.WPRE* (UCL Great Ormond Street Institute of Child Health, London, UK) and packaging constructs *pCMVdR8.74* and *pMDG.2* (Addgene plasmid repository, Cambridge, MA, USA), as described previously [28]. The *pRRL* self-inactivating vector plasmid was previously constructed at UCL Great Ormond Street Institute of Child Health [6], and contains the *GLA* transgene which encodes mature human α-gal-A. The transgene is regulated by the human phosphoglycerate kinase (hPGK) promoter which allows ubiquitous expression. A central polypurine tract (*cPPT*) allows reverse transcription and formation of a central overlap of DNA that increases transduction efficiency and may promote nuclear import. A modified woodchuck hepatitis virus post-transcriptional regulatory element (*WPRE*) increases transcription of unspliced RNA and transgene expression [45]. Similarly, lentivirus containing the reporter gene *GFP* as a control was generated using vector plasmid *pRRLSIN.cPPT.PGK-GFP.WPRE* (Addgene plasmid #12252, gifted by Professor D Trono (Ecole Polytechnique Federale de Lausane, Switzerland)). Culture media were collected 48 and 72 h post-transfection and centrifuged at 98,000 g for 2 h. The concentrated virus was titred on Jurkat cells (ATCC, Teddington, UK) by qPCR.

### 4.3. Transduction of Jurkat Cells

2 × 10^5^ Jurkats were seeded in 1.5 mL RPMI (Life, Paisley, UK) + 10% FBS (Sigma, Poole, UK) and various doses of lentivirus containing either *GLA* or *GFP* (10 μL–1 μL). The 6-well plates were incubated for three days; after one day, a further 1.5 mL fresh culture media was added to each well. All cells were washed three times in Dulbecco’s PBS (Life, Paisley, UK) to thoroughly remove virus before assay or further experiments.

### 4.4. Quantitative PCR

Absolute quantification of lentivirus WPRE and genomic β-actin sequences were measured against a plasmid pBTW2L standard curve (kindly provided by Dr Conrad Vink, UCL GOSICH, London, UK) on the Bio-Rad c1000 Touch thermo-cycler (Bio-Rad, Hertfordshire, UK) using the following conditions: 50 °C for 2 min, 95 °C for 10 min, then 40 cycles of 95 °C for 15 s and 60 °C for 1 min. Amplification of β-actin and WPRE sequences were used to calculate cell number and viral copy number respectively using Bio-Rad CFX manager software (Available online: www.bio-rad.com). 

### 4.5. Cell Toxicity

Approximately 3 × 10^5^ *GLA*-Jurkats were seeded and the number of live and dead cells was counted every 24 h for three days as follows. Cell viability was calculated at each timepoint as the percentage of all cells alive. Cells in media suspension were stained with trypan blue solution at 1:1 and counted using a haemocytometer (Camlab, Cambridge, UK). This experiment was carried out in triplicate and is based on the principle that live cells possess intact cells membranes and exclude the dye.

### 4.6. Cell Lysis

For lysosomal enzyme activity assays, Jurkats were collected and re-suspended in distilled H_2_O and lysed by sonication using a Soniprep 150 (MSE, Heathfield, UK) for 10 s at 6 μm amplitude. For mitochondrial enzyme activity assays, cells were exposed to three fast freeze-thaw cycles using dry ice in methanol and 37 °C water bath. Protein concentration in cell lysate was measured using the BCA method [46] on the Cecil 2040 spectrophotometer against standard 1mg/mL bovine serum albumin in water (Sigma, Poole, UK).

### 4.7. Lysosomal Enzyme Activity Assays

Intracellular total α-gal-A [47], and extracellular total α-gal-A activity [48] were both measured using the fluorogenic synthetic substrate 4-methylumbelliferyl-α-D-galactopyranoside (4MUG) (Melford, Ipswich, UK), i.e., with the degree of florescence produced being directly proportional to enzyme activity. The B isoform, which possesses residual α-galactosidase activity, was competitively inhibited using N-acetyl-D-galactosamine (NAGA) (Sigma, Poole, UK) [49]. In addition, the activity of some other lysosomal enzymes was determined to assess the specificity of the effects of gene transfer upon α-galactosidase activity and release. These included β-galactosidase activity which was measured in cultured cells as described by [50], using the substrate 4-methylumbelliferyl-β-D-galactopyranoside (Sigma, Poole, UK). N-acetyl-galactosaminidase activity was measured in cultured cells using the synthetic substrate 4-methylumbelliferyl-α-N-acetyl-galactosaminide (Sigma, Poole, UK) [51]. 

Total β-hexosaminidase activity was measured using the substrate 4-methylumbelliferyl-2-acetaminido-2-deoxy-β-D-glucopyranoside (Melford, Ipswich, UK). In cultured cells the method was based upon that of Brett et al. [52]; extracellular activity was measured based upon the method described by O’Brien et al. [53]. In all assays, fluorescence was quantified using the Perkin Elmer LS55 fluorimeter (Perkin Elmer, Seer Green, UK) calibrated against a 1nmol 1,4-methylumbelliferone (4MU) (Sigma, Poole, UK) standard.

### 4.8. Kinetic Studies

Alpha-gal-A activity was measured in wild-type and *GLA*-Jurkats over a range of final substrate concentrations from 0.25 mM to 9 mM. Higher final concentrations (15 mM and 30 mM) were attempted but 4MUG precipitated from solution at 37 °C. A best-fit substrate saturation curve was plotted through the data using least-squares non-linear regression analysis obeying the Michaelis-Menton equation using Prism 5.01v software (GraphPad Software, SanDiego, CA, USA) (https://www.graphpad.com/guides/prism/7/curve-fitting/index.htm?reg_michaelis_menten_enzyme.htm).

### 4.9. Cross Correction Studies

Donor media, collected from *GLA*-Jurkats after 2 days incubation at 37 °C, 5% CO_2_, were collected for assay. Donor media were mixed 3:1 with fresh complete RPMI to provide sufficient nutrients for cell growth. This is consistent with the method used by Neufeld and colleagues [54]. The mixed donor media were then incubated with recipient cells for two days. To take account of any extracellular enzyme degradation during incubation, mixed donor media were also incubated without recipient cells present. 

### 4.10. Mitochondrial Complex I Inhibition

Irreversible inhibition of mitochondrial complex I was achieved by the use of 100 nM rotenone (Sigma, Poole, UK) that was prepared in ethanol (>99.8%) as we have previously described [41]. Rotenone (100 nM final concentration) was added to 10 mL RPMI + 10%FBS containing 5 × 10^5^ wild-type Jurkat cells. As control, 5 × 10^5^ Jurkats were grown in the same volume of media and ethanol (>99.8%). The cross correction studies were repeated as above. In this way, the effect of rotenone alone was identified on cell transduction, enzyme release by *GLA*-Jurkats and uptake by recipient cells. Area under curves was calculated using Prism 5.01v (GraphPad Software, San Diego, CA, USA).

### 4.11. Mitochondrial Complex I and Citrate Synthase Activity

Rotenone sensitive mitochondrial complex I (NADH-ubiquinone oxidoreductase) activity was assessed, in cell homogenates (derived from washed cells), by measuring the oxidation of NADH at 340 nm using CoQ1 as the electron acceptor as described previously by [55]. Since the activity of complex I in whole cell homogenate may vary with mitochondrial content between cells, the activity of the mitochondrial marker enzyme, citrate synthase, was determined [56] to account for mitochondrial enrichment [57,58]. Absorbance was measured using a Uvikon XL spectrophotometer (NorthStar Scientific, Leeds, UK). 

### 4.12. Statistics

Data are summarised as mean ± standard deviation (SD). The n value is defined as the number of repeat, independent cell culture preparations analysed. In cell-toxicity experiments, the number of live and dead cells in each preparation was counted in duplicate. In enzyme assays, each preparation was measured in duplicate. Virus copy number of each preparation was measured in triplicate. The difference between two data-sets was analysed by Student’s t-test; difference between three or more data-sets was measured by one-way analysis of variance (ANOVA) and Bonferroni’s post hoc tests. An F test was performed to demonstrate equal variances. Cell toxicity data were analysed by two-way ANOVA to take account of both duration of incubation and virus dosage. Scatter plots were analysed by linear regression (R^2^) if significant correlation was found. In all cases, *p* < 0.05 was considered to be significant. Before statistical analysis of percentage and ratio data, values were inverse sine square root transformed. Statistical testing was performed using Prism 5.01v (GraphPad Software, San Diego, CA, USA).

## Figures and Tables

**Table 1:** Kinetic properties of α-gal-A from wild-type Jurkats and *GLA* over expressing Jurkats using the fluorogenic synthetic substrate, 4-methylumbelliferyl-α-D-galactopyranoside (4MUG).

Kinetic Parameter	Wild Type	0.4 vg/cell	1.5 vg/cell	2.0 vg/cell	1.8 vg/cell (Dilute)
Km (mM)	1.57 ± 0.11	1.52 ± 0.13	2.43 ± 0.12	2.30 ± 0.10	2.53 ± 0.29
Vmax (nmol/hr)	1.95 ± 0.04	8.31 ± 0.02	67.30 ± 1.12	82.1 ± 1.20	1.17 ± 0.06

## Abbreviations

FD	Fabry Disease
α-gal-A	α-galactosidase-A
Gb3	globotriaoslyceramide
ERT	Enzyme Replacement Therapy
GFP	Glial Fibrillary Acid Protein
